# Contrasting roles for IgM and B-cell MHCII expression in *Brucella abortus* S19 vaccine-mediated efficacy against *B. melitensis* infection

**DOI:** 10.1128/msphere.00750-23

**Published:** 2024-02-13

**Authors:** Mostafa F. N. Abushahba, Alexis S. Dadelahi, Bárbara Ponzilacqua-Silva, Charles R. Moley, Jerod A. Skyberg

**Affiliations:** 1 Department of Veterinary Pathobiology, College of Veterinary Medicine, University of Missouri, Columbia, Missouri, USA; 2 Laboratory for Infectious Disease Research, University of Missouri, Columbia, Missouri, USA; 3 Department of Zoonoses, Faculty of Veterinary Medicine, Assiut University, Assiut, Egypt; University of Florida, USA

**Keywords:** brucellosis, zoonoses, S19, RB51, vaccine, B cell, IgM, complement

## Abstract

Brucellosis, caused by the bacterium *Brucella*, poses a significant global threat to both animal and human health. Although commercial live *Brucella* vaccines including S19, RB51, and Rev1 are available for animals, their unsuitability for human use and incomplete efficacy in animals necessitate the further study of vaccine-mediated immunity to *Brucella*. In this study, we employed *in vivo* B-cell depletion, as well as immunodeficient and transgenic mouse models, to comprehensively investigate the roles of B cells, antigen uptake and presentation, antibody production, and class switching in the context of S19-mediated immunity against brucellosis. We found that antibody production, and in particular secretory IgM plays a protective role in S19-mediated immunity against virulent *Brucella melitensis* early after the challenge in a manner associated with complement activation. While T follicular helper cell deficiency dampened IgG production and vaccine efficacy at later stages of the challenge, this effect appeared to be independent of antibody production and rather was associated with altered T-cell function. By contrast, B-cell MHCII expression negatively impacted vaccine efficacy at later timepoints after the challenge. In addition, B-cell depletion after vaccination, but before the challenge, enhanced S19-mediated protection against brucellosis, suggesting a deleterious role of B cells during the challenge phase. Collectively, our findings indicate antibody production is protective, while B-cell MHCII expression is deleterious, to live vaccine-mediated immunity against brucellosis.

**IMPORTANCE:**

*Brucella* is a neglected zoonotic pathogen with a worldwide distribution. Our study delves into B-cell effector functions in live vaccine-mediated immunity against brucellosis. Notably, we found antibody production, particularly secretory IgM, confers protection against virulent *Brucella melitensis* in vaccinated mice, which was associated with complement activation. By contrast, B-cell MHCII expression negatively impacted vaccine efficacy. In addition, B-cell depletion after vaccination, but before the *B. melitensis* challenge, enhanced protection against infection, suggesting a detrimental B-cell role during the challenge phase. Interestingly, deficiency of T follicular helper cells, which are crucial for aiding germinal center B cells, dampened vaccine efficacy at later stages of challenge independent of antibody production. This study underscores contrasting and phase-dependent roles of B-cell effector functions in vaccine-mediated immunity against *Brucella*.

## INTRODUCTION

Brucellosis, caused by the Gram-negative bacterium *Brucella*, stands as a significant zoonotic disease that carries extensive implications for global human and animal health. Despite its profound impact, it remains a disease that has not garnered sufficient attention (1). This facultative intracellular pathogen has adeptly broadened its reservoirs to encompass a variety of domestic and wildlife animals (2), rendering its eradication a formidable challenge. Brucellosis affects an estimated 2.1 million humans annually worldwide, with the pathogen also infecting over 300 million cattle out of the 1.4 billion total cattle population (3, 4). In animals, brucellosis primarily results in abortion and other manifestations such as retained placentas, decreased milk yield, and decreased fertility (5). In humans, acquiring the disease can occur through various routes, including contact with infected animals, consumption of contaminated animal products, and inhalation of airborne agents (6). The impact of brucellosis on human health is wide ranging, spanning from the characteristic undulant fever to more debilitating effects such as arthritis, orchitis, hepatitis, and endocarditis (7–11).

The lack of effective treatments for animal brucellosis and the high cost of treating infected humans (5, 12) emphasize the necessity for robust disease prevention. Current vaccines such as S19, RB51, and Rev1 exhibit around 70% efficacy against animal brucellosis (13–15), underscoring the need for further research on vaccine-mediated immunity.

Various investigations, including our own, have found that B-cell deficiency renders mice more resistant to brucellosis (16–18), indicating a detrimental role of B cells in primary infection. This negative role imposed by B cells on resistance to *Brucella* is antibody independent (16, 18). By contrast, in a model of secondary brucellosis, a prior study has indicated an indispensable role for B cells in controlling infection (19, 20). Other studies have provided evidence that passive antibody transfer from infected or immunized animals protects naïve mice from brucellosis (21–26). However, in bovine, high levels of IgG1 and IgG2 antibodies following infection were found to hinder complement-mediated killing of *B. abortus in vitro* presumably due to the “prozone effect” and there is generally thought to be a lack of positive correlation between antibody titers and resistance to brucellosis (27).

The S19 vaccine is cross-protective against *B. melitensis* in laboratory animals and dairy cattle (28–30). As we previously investigated B-cell effector functions during primary infection with *B. melitensis* (18, 31) here we employed a S19 vaccination/*B. melitensis* challenge model to determine the role of B-cell effector functions in vaccine-mediated immunity against *Brucella*. Our comprehensive investigation employed a range of immunodeficient and transgenic mouse models, along with depletion assays, to explore the contributions of B-cell and T follicular helper cell responses to vaccine-mediated immunity against brucellosis.

## RESULTS

### B cells exert a phase-dependent detrimental impact on S19 vaccine-mediated efficacy against brucellosis

**Fig 1:**
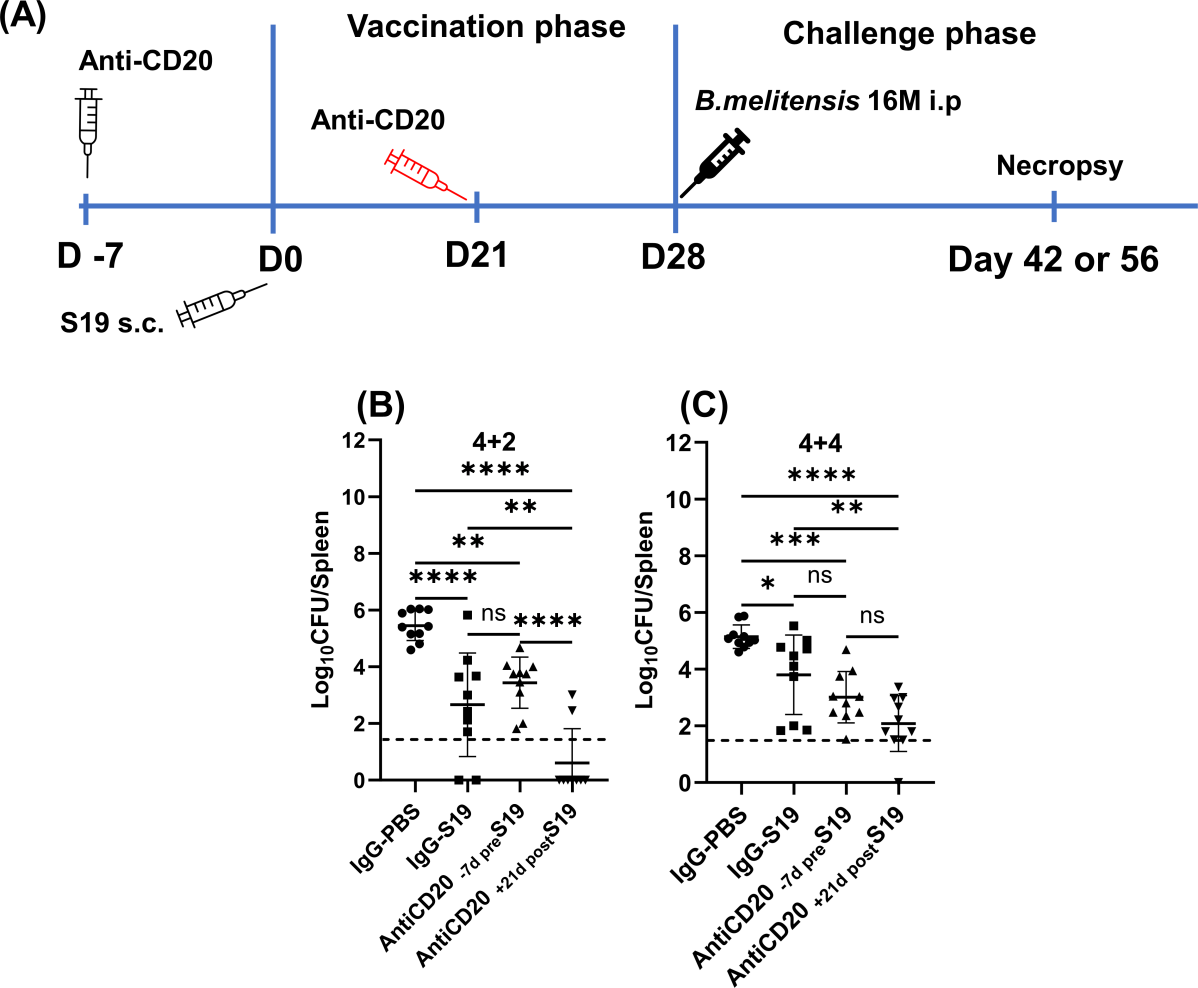
Impact of temporal B-cell deficiency on S19-mediated efficacy against brucellosis. (**A**) Schematic of the B-cell depletion, vaccination, and challenge strategy employed. Mice were s.c. vaccinated with 2 × 10^5^ CFUs of S19 vaccine and were challenged i.p. 4 weeks later with 1 × 10^5^ CFUs of *B. melitensis* 16M. For B-cell depletion, an anti-CD20 antibody was injected i.p to WT mice either at 7 days before vaccination (referred to the figures as Anti-CD20 _-7pre_S19) or 21 days after vaccination (referred in the figures as Anti-CD20 _+21post_S19). (**B**) Splenic *B. melitensis* burdens in B-cell depleted and IgG-treated vaccinated WT mice (*n* = 9–10 mice/per group) measured after 4 weeks of vaccination and 2 weeks of challenge (4 + 2). (**C**) Splenic *B. melitensis* burdens in B-cell depleted and IgG-treated vaccinated WT mice (*n* = 10 mice/per group) measured after 4 weeks of vaccination and 4 weeks of challenge (4 + 4). Data in (**C**) are combined from two experiments. Dashed lines indicate the limit of detection.

Previously, we found that B-cell deficiency or depletion enhanced the resistance of mice to primary infection with *B. melitensis* (18, 31). However, in secondary brucellosis, prior research utilizing B-cell-deficient mice, where infected animals were treated with antibiotics and then rechallenged with *Brucella,* has suggested somewhat variable roles on the contributions of B cells which might be related to the route of infection or time post-challenge when control of infection was evaluated (19, 32). Thus, we employed a vaccination/challenge approach (33), combined with B-cell depletion, to evaluate the impact of temporal B-cell deficiency on vaccine-mediated immunity against brucellosis. B cells were depleted either 7 days prior to vaccination or 21 days post-vaccination (one week before challenge, Fig. 1A) with anti-CD20 antibody. B-cell depletion in blood was found to be ≥90% effective at the time of challenge (Fig. S1A) and significantly reduced anti-*Brucella* IgM and IgG levels (Fig. S1B and C). B-cell depletion prior to vaccination raised splenic *B. melitensis* burdens 2 weeks post-challenge, though this difference was not significant (Fig. 1B). Conversely, depleting B cells from mice at day 21 after vaccination (1 week before challenge) significantly reduced splenic burdens compared to isotype treated mice at this timepoint (Fig. 1B). This indicates that the presence of B cells during vaccination, followed by their removal before challenge is advantageous in protecting against virulent *Brucella* infection. Similarly, after 4 weeks of vaccination and 4 weeks post-challenge, we again observed that B-cell depletion prior to the challenge improved resistance to brucellosis (Fig. 1C), further validating the deleterious role of B cells during the challenge phase. Taken together, these findings demonstrate that B cells have time-dependent roles in the setting of S19 vaccination and *Brucella* infection.

### B cells are deleterious during the *Brucella* challenge phase in an MHCII-dependent manner

**Fig 2:**
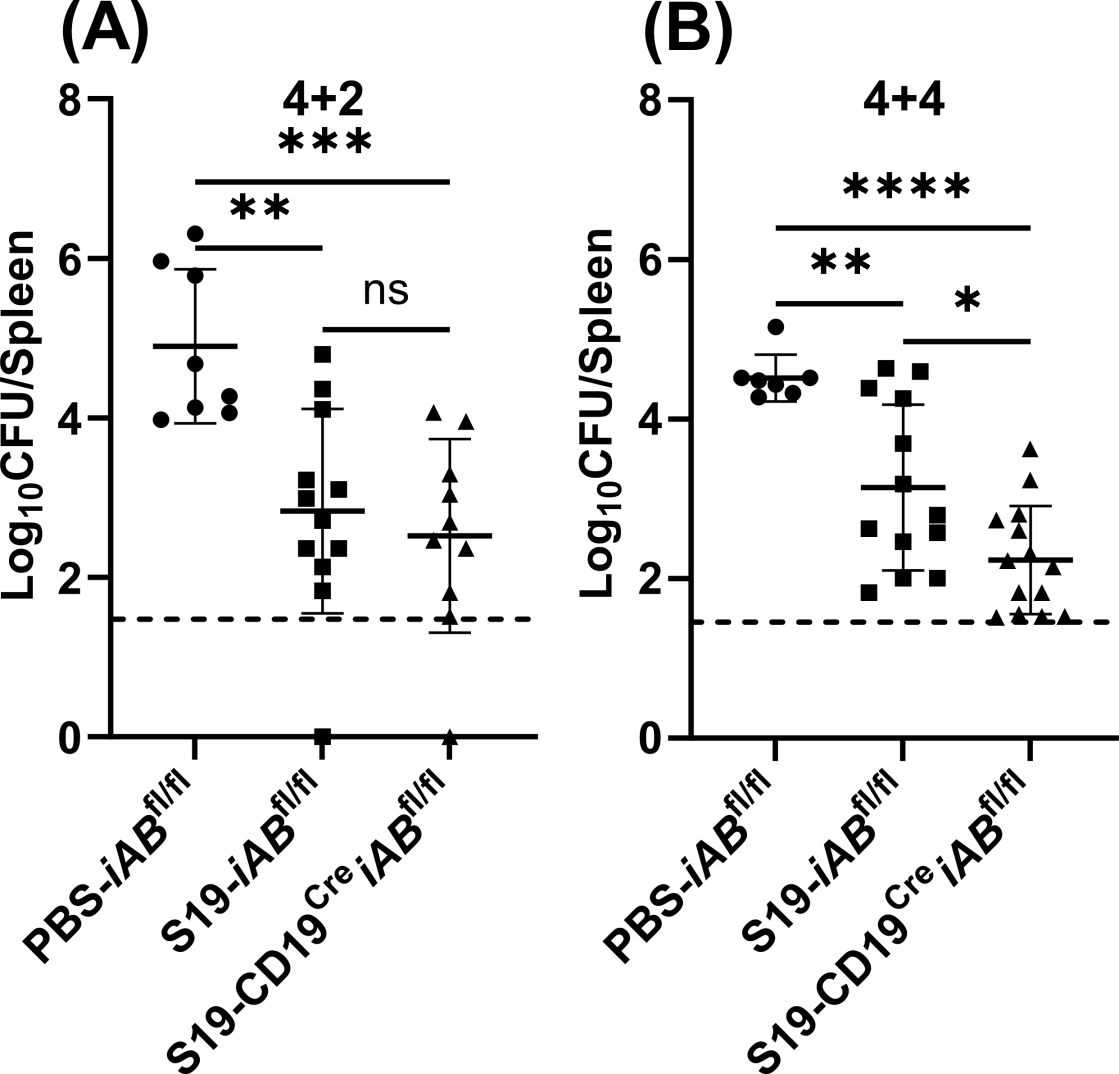
B-cell MHCII expression is deleterious to S19-mediated efficacy. Mice were s.c vaccinated with 2 × 10^5^ CFUs of S19 and were challenged 4 weeks later with 1 × 10^5^ CFUs i.p. of *B. melitensis* 16M. (**A**) Splenic *B. melitensis* burdens in CD19^Cre^*iAB*^fl/fl^ and *iAB*^fl/fl^ mice (*n* = 8–12 mice/per group) measured after 4 weeks of vaccination and 2 weeks of challenge (4 + 2). (**B**) Splenic *B. melitensis* burdens in CD19^Cre^*iAB*^fl/fl^ and *iAB*^fl/fl^ mice (*n* = 7–14 mice/per group) measured after 4 weeks of vaccination and 4 weeks of challenge (4 + 4). Data are combined from two experiments. Dashed lines indicate the limit of detection.

We previously demonstrated B-cell MHCII expression enhances susceptibility to primary brucellosis (18). Therefore, we utilized CD19^Cre^*iAB*^fl/fl^ mice that specifically lack B-cell MHCII expression (31) to determine whether the detrimental effect exerted by B cells during the challenge following S19 vaccination is contingent on their antigen presentation capability. Similar to what we observed during primary infection (31), anti-*Brucella* IgG levels were reduced in CD19^Cre^*iAB*^fl/fl^ mice following vaccination with S19 (Fig. S1D). After 4 weeks of vaccination and 2 weeks of challenge, we observed similar levels of *B. melitensis* in *iAB*^fl/fl^ and CD19^Cre^*iAB*^fl/fl^ mice, which were reduced ~90- to 180-fold relative to PBS-treated *iAB*^fl/fl^ control mice (Fig. 2A). However, after 4 weeks of vaccination and 4 weeks of challenge, we observed a significant reduction in the splenic burden of S19-vaccinated B-cell MHCII-deficient mice in relation to vaccinated control animals (Fig. 2B), indicating B-cell MHCII expression is detrimental to S19-mediated efficacy at later stages after challenge. Taken together, these observations suggest MHCII expression on B cells undermines the effectiveness of the S19 vaccine against brucellosis.

### BCR specificity has a dispensable role in S19-mediated efficacy against brucellosis

**Fig 3:**
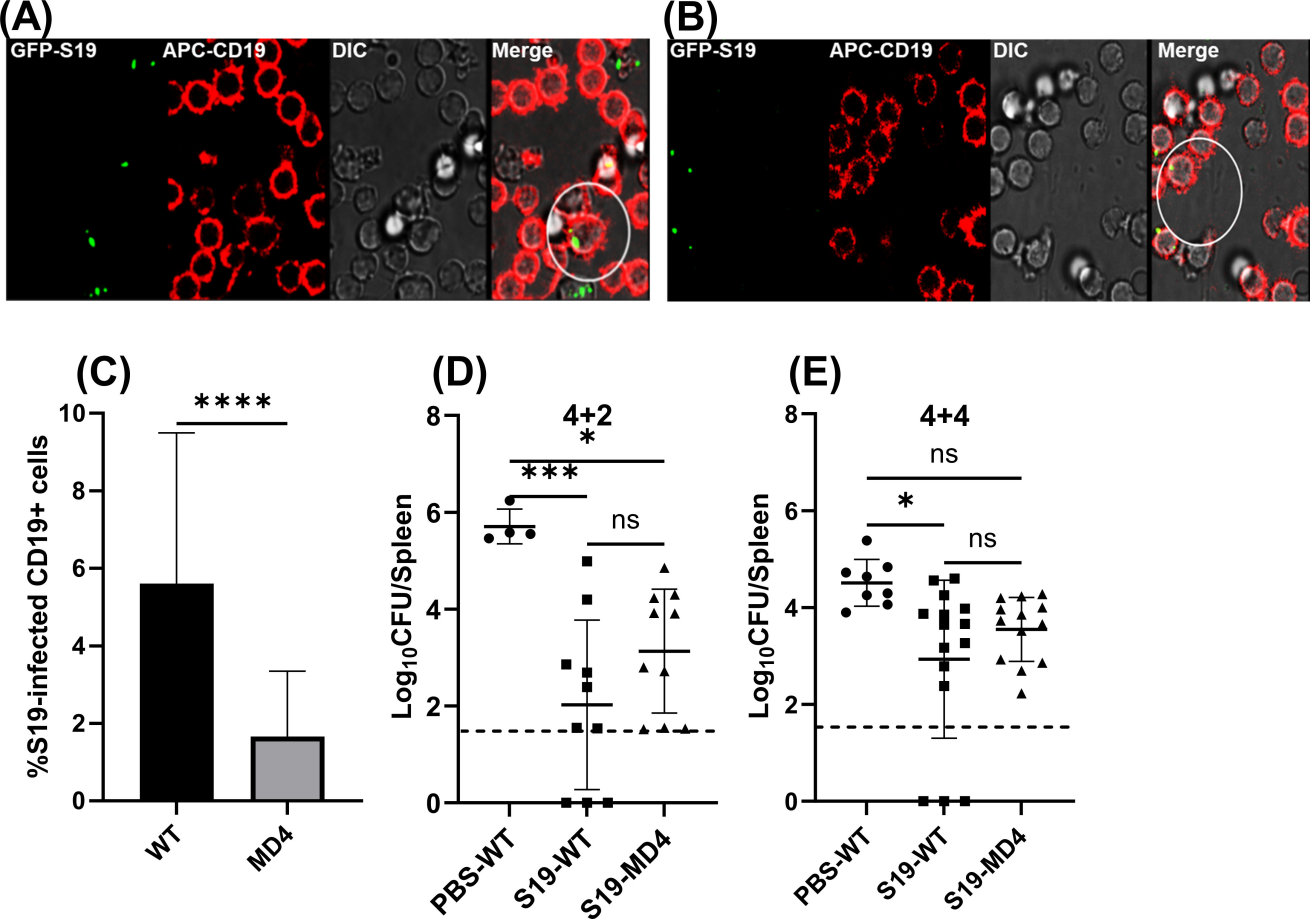
BCR specificity impacts S19 uptake by B cells but does not influence S19-mediated efficacy against brucellosis. (**A–C**) Confocal images (**A and B**) and quantification (**C**) of GFP-S19 in B cells from WT (**A**) or MD4 (**B**) mice in *vitro* following 4.5 hours of infection (MOI = 500). (D & E) Mice were s.c vaccinated with 2 × 10^5^ CFUs of S19 and were challenged 4 weeks later with 1 × 10^5^ CFUs i.p. of *B. melitensis* 16M. (**D**) Splenic *B. melitensis* burdens in MD4 and WT mice (*n* = 4–10 mice/per group) were measured after 4 weeks of vaccination and 2 weeks of challenge (4 + 2). (**E**) Splenic *B. melitensis* burdens in MD4 and WT mice (*n* = 8–15 mice/per group) were measured after 4 weeks of vaccination and 4 weeks of challenge (4 + 4). Data in (**E**) are combined from two experiments. Dashed lines indicate the limit of detection.

BCR-mediated mechanisms of antigen uptake are 100–1,000 times more efficient in inducing cognate T-cell activation through MHCII compared to BCR-independent mechanisms of antigen uptake (34). In light of our finding that B-cell MHCII expression diminishes S19 vaccine-mediated protection (Fig. 2B), we investigated the influence of BCR specificity on S19-mediated immunity against *Brucella* by employing MD4 mice, in which ~90% of B cells express a BCR specific for the irrelevant antigen hen egg lysozyme (HEL) (35). First, we compared the ability of B cells from WT and MD4 mice to uptake *Brucella* by exposing splenocytes obtained from WT or MD4 mice to GFP-expressing S19 *in vitro* and employing confocal microscopy to visualize the presence of bacteria within B cells. Our results revealed that B cells from MD4 mice can uptake GFP-S19, albeit at a level ~3 times lower than B cells from WT mice (Fig. 3A through C), suggesting BCR specificity plays a significant role in the uptake of S19.

Consequently, we investigated whether BCR specificity affects S19 efficacy by vaccinating MD4 and WT littermate control mice and comparing splenic bacterial loads at 2 and 4 weeks post-challenge. Surprisingly, at both times post-challenge, the lack of BCR specificity to *Brucella* antigen did not significantly alter *B. melitensis* levels in S19-vaccinated mice (Fig. 3D and E). These findings could suggest that BCR specificity does not mediate the detrimental effects of B-cell MHCII expression on vaccine- mediated *Brucella*. However, BCR specificity also mediates antibody specificity, and we previously found impaired anti-*Brucella* antibody production in MD4 mice (31). Thus, we sought to determine whether impaired *Brucella*-specific antibody production in MD4 mice could mask a potential deleterious role of BCR specificity on vaccine-mediated immunity against *Brucella*.

### Vaccine-elicited IgM and complement are crucial to control the early dissemination of *Brucella*

**Fig 4:**
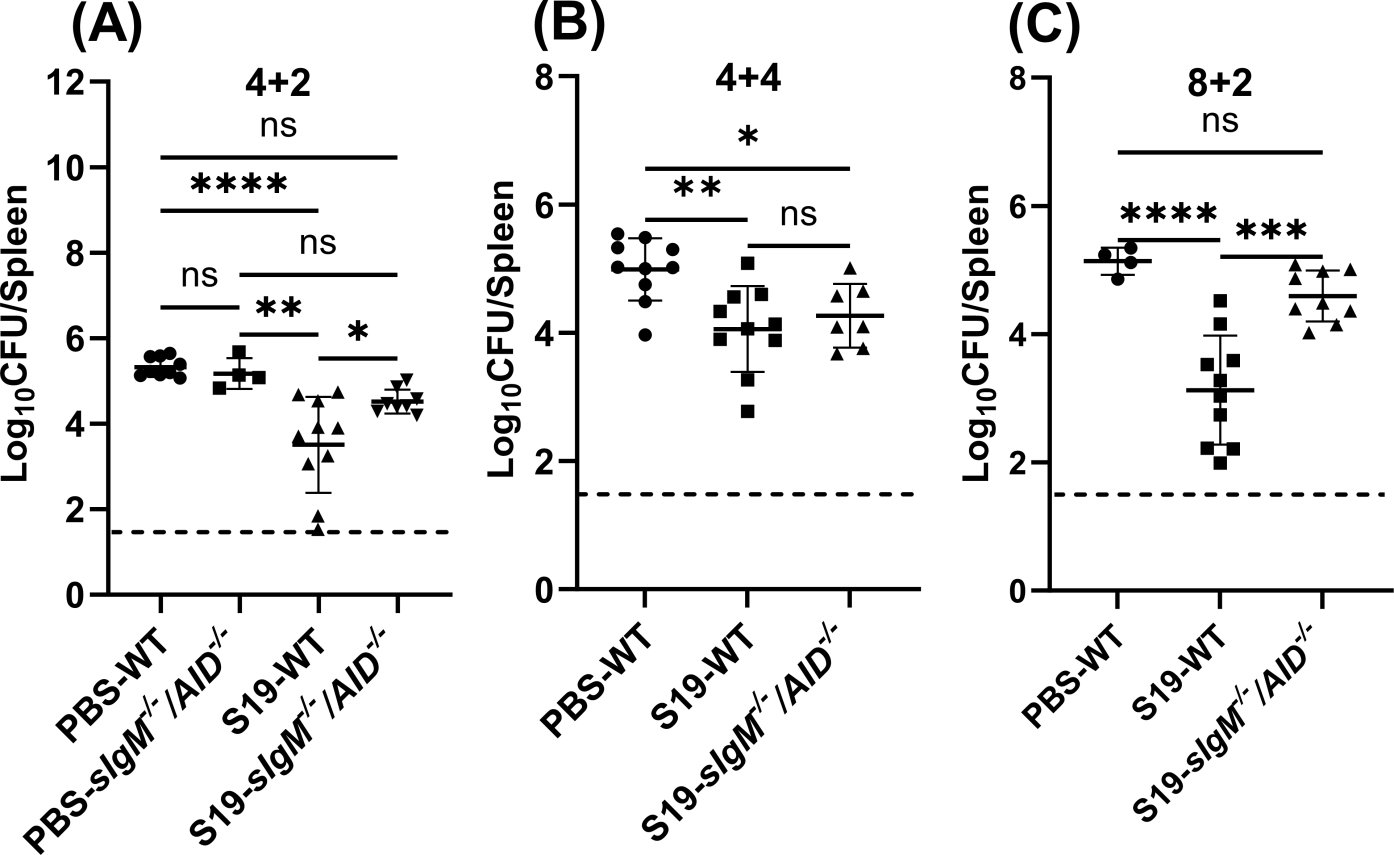
Vaccine elicited antibodies are necessary for protecting against brucellosis at the early stage of infection. Mice were s.c vaccinated with 2 × 10^5^ CFUs of S19 and were challenged 4 or 8 weeks later with 1 × 10^5^ CFUs i.p. of *B. melitensis* 16M. (**A**) Splenic *B. melitensis* burdens in *sIgM^−/−^/AID*^−/−^ and WT mice (*n* = 4–10 mice/per group) measured after 4 weeks of vaccination and 2 weeks of challenge (4 + 2). (**B**) Splenic *B. melitensis* burdens in *sIgM^−/−^/AID*^−/−^ and WT mice (*n* = 7–10 mice/per group) measured after 4 weeks of vaccination and 4 weeks of challenge (4 + 4). (**C**) Splenic *B. melitensis* burdens in *sIgM^−/−^/AID*^−/−^ and WT mice (*n* = 4–10 mice/per group) measured after 8 weeks of vaccination and 2 weeks of challenge (8 + 2). Data in (**B**) are combined from two experiments.

Passive transfer of immune sera from infected or vaccinated animals has indicated antibodies can protect recipients against *Brucella* (21–26). In addition, by comparing results in B-cell-deficient mice and mice unable to undergo class switching others have suggested a protective role of IgM in secondary *Brucella* infection (19). By contrast, in primary infection, we found an inability to secrete antibodies does not affect the control *of B. melitensis* within the first month after infection (Fig. S2A) ( 31). To clarify the role of humoral immunity in vaccine-mediated immunity against *Brucella*, we used *sIgM^−/−^/AID^−/−^* mice which express a polyclonal B-cell receptor but are unable to secrete IgM or class-switched antibodies (Fig. S2B and C) (36). Our findings revealed that 2 weeks post-challenge, S19-vaccinated *sIgM^−/−^/AID^−/−^* mice, vaccinated 4 weeks or 8 weeks before the challenge, had significantly higher splenic loads relative to WT mice (Fig. 4A and C). By contrast, we observed that the absence of antibodies had no impact on the efficacy of the S19 vaccine at 4 weeks post-challenge (Fig. 4B). These findings indicate that anti-S19 secretory antibodies play a crucial role in limiting the early dissemination of virulent *Brucella;* however, the role of antibodies diminishes as infection advances.

**Fig 5:**
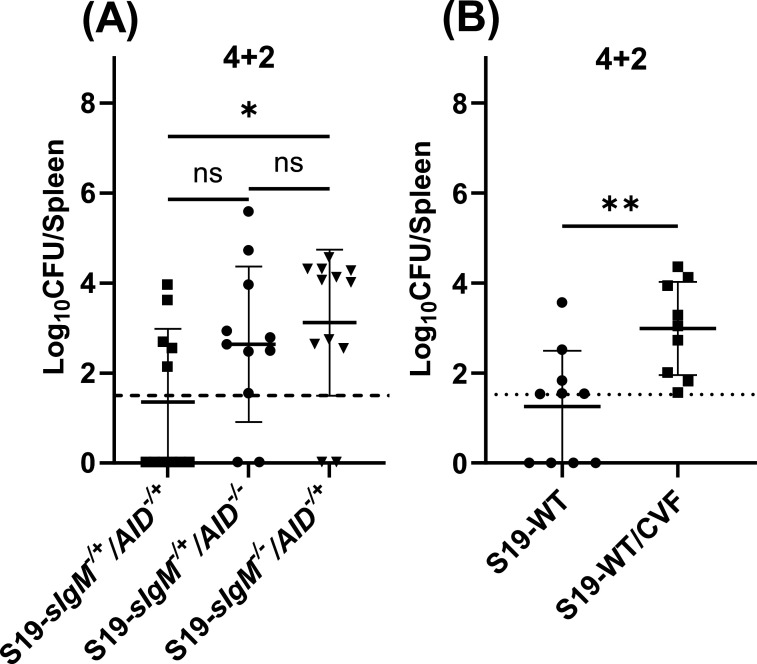
IgM and complement contribute to S19-mediated efficacy against brucellosis. Mice were s.c vaccinated with 2 × 10^5^ CFUs of S19 and were challenged 4 weeks later with 1 × 10^5^ CFUs i.p. of *B. melitensis* 16M. (**A**) Splenic *B. melitensis* burdens in mice lacking the ability to secrete IgM (*sIgM^−/−^/AID^−/+^*), class-switched antibodies (*sIgM^−/+^/AID*^−/−^), and heterozygous control *sIgM^−/+^/AID^−/+^* mice (*n* = 11–12 mice/per group) measured after 4 weeks of vaccination and 2 weeks of challenge (4 + 2). (**B**) WT mice (*n* = 9–10 mice/per group) were s.c vaccinated with 2 × 10^5^ CFUs of S19 and challenged i.p. 4 weeks later with 1 × 10^5^ CFUs of *B. melitensis* 16M. Some mice were also depleted of complement with cobra venom factor (CVF). *B. melitensis* was measured after 4 weeks of vaccination and 2 weeks of challenge (4 + 2). Data in (**A**) are combined from two experiments. Dashed lines indicate the limit of detection.

We proceeded to investigate the specific contribution of IgM and class-switched antibodies by generating mice unable to secrete class-switched antibodies (*sIgM^−/+^/AID^−/−^*) or IgM (*sIgM^−/−^/AID^−/+^*). We then evaluated the ability of S19 to protect these strains against *Brucella*. Serological analysis (Fig. S2C and D) confirmed IgM and IgG production was ablated in *sIgM^−/−^/AID^−/+^* and *sIgM^−/+^/AID^−/−^* mice, respectively. Bacterial levels trended higher in S19-vaccinated mice unable to produce class-switched antibodies relative to controls, though this difference was not statistically significant (Fig. 5A). However, vaccine efficacy was significantly impaired in mice unable to secrete IgM (Fig. 5A) indicating a pivotal role of IgM in vaccine-mediated immunity against *Brucella*. A key function of IgM is the activation of complement (37). Therefore, we depleted S19 vaccinated mice of complement by treating them with cobra venom factor (CVF). As revealed in Fig. 5B, complement depletion in vaccinated mice significantly increased splenic *B. melitensis* burdens 2 weeks post-challenge relative to controls, indicating a crucial role of complement in vaccine-mediated immunity against brucellosis.

### *Bcl6* deficiency is associated with impaired vaccine efficacy and altered CD4^+^ T-cell transcriptomic profile

**Fig 6:**
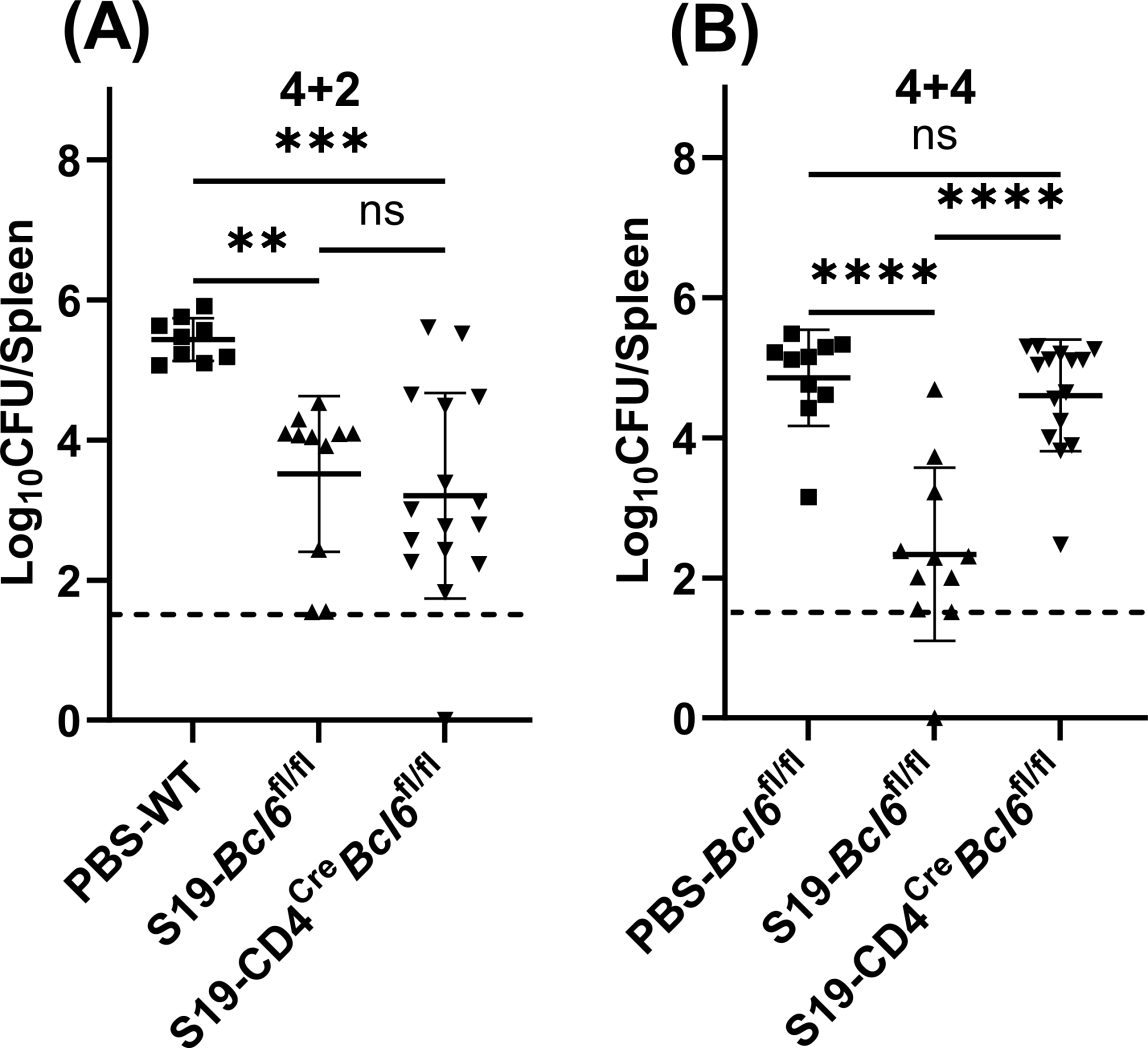
CD4^+^ T-cell *Bcl6* deficiency is associated with impaired control of *Brucella* in S19-vaccinated mice. (**A**) Splenic *B. melitensis* burdens in WT, CD4^cre^*Bcl6*^fl/fl^, and *Bcl6*^fl/fl^ mice (*n* = 9–16 mice/per group) measured after 4 weeks of vaccination and 2 weeks of challenge (4 + 2). (**B**) Splenic *B. melitensis* burdens in CD4^cre^*Bcl6*^fl/fl^ and *Bcl6*^fl/fl^ mice (*n* = 10–15 mice/per group) measured after 4 weeks of vaccination and 4 weeks of challenge (4 + 4). Data are combined from two experiments. Dashed lines indicate the limit of detection.

Our investigation on the role of antibodies was furthered by employing CD4^Cre^*Bcl6*^fl/fl^ mice, in which *Bcl6* deletion in CD4^+^ T cells results in T follicular helper cell (T_FH_) deficiency. Isotype switching and germinal center responses are also impaired in mice lacking Bcl6 expression in CD4^+^ T cells (38). Analysis of antibody levels 4 weeks post-vaccination indicated T_FH_ deficient mice exhibited comparable anti-*Brucella* IgM levels but significantly dampened anti-*Brucella* IgG levels relative to their vaccinated *Bcl6*^fl/fl^ counterparts (Fig. S3A and B). Interestingly, at 2 weeks post-challenge, the absence of the T_FH_ did not significantly influence the efficacy of S19 vaccination (Fig. 6A). This reinforced the idea that the protective role conferred by humoral immunity is predominantly linked to IgM rather than IgG. However, as infection progressed to the 4-week stage, the efficacy of S19 was significantly diminished in T_FH_-deficient mice compared to *Bcl6*^fl/fl^ mice (Fig. 6B). While *Bcl6* deficiency in CD4^+^ T cells results in T_FH_ deficiency, impaired germinal center formation, and defects in class-switched antibody production (39), at 4 weeks post-challenge an inability to secrete antibodies did not affect S19-mediated immunity against *B. melitensis* (Fig. 4B). Therefore, we investigated the effects of *Bcl6* deficiency on the function of CD4^+^ T cells in the context of vaccination and *Brucella* challenge.

### *Bcl6* deficiency is associated with altered CD4^+^ T-cell transcriptomic profile

**Fig 7:**
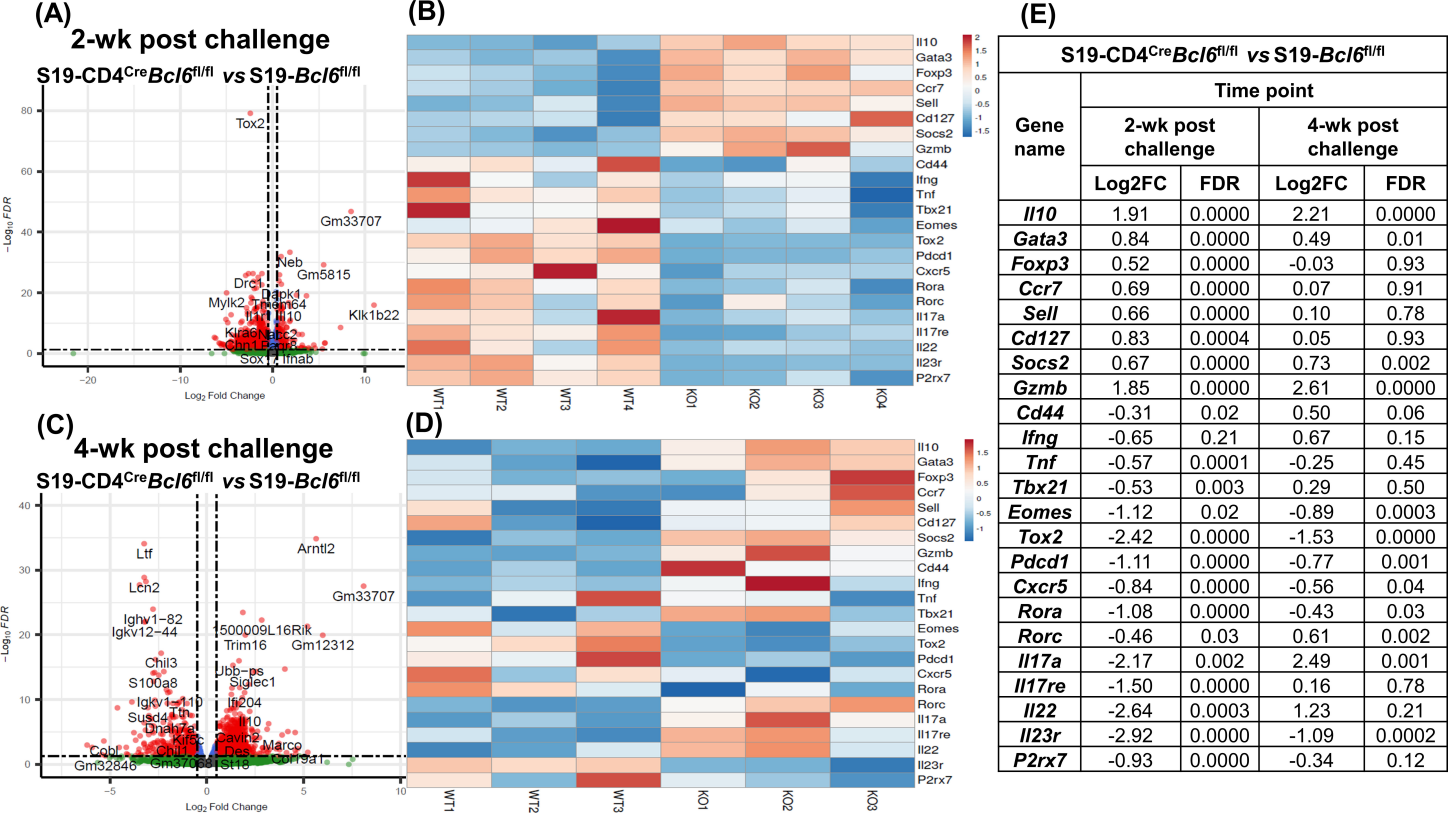
*Bcl6* deficiency is associated with an altered CD4^+^ T-cell transcriptomic profile. Mice were s.c vaccinated with 2 × 10^5^ CFUs of S19 vaccine and were challenged 4 weeks later with 1 × 10^5^ CFUs i.p. of *B. melitensis* 16M. On 2 and 4 weeks post-infection, CD4^+^ T cells were purified from spleens and RNA was extracted for RNA-Seq analysis. (A, **C**) Volcano plot showing genes with altered gene expression in CD4^Cre^*Bcl6*^fl/fl^ and *Bcl6*^fl/fl^ mice CD4^+^ T cells at 2 weeks (**A**) or 4 weeks (**C**) post-challenge in previously vaccinated mice. The horizontal dashed line indicates a false discovery rate (FDR) of 0.05, and genes outside the vertical dashed lines have an absolute log2 fold change of >0.5. (B & D) Heat map of 23 selected genes whose expression was altered by CD4^+^ T-cell *Bcl6* deficiency after vaccination and either 2 (**B**) or 4 (**D**) weeks post*-B. melitensis* challenge. (**E**) Table depicting a comparison of 23 selected genes altered in the context of CD4^+^ T-cell *Bcl6* deficiency at 2 and 4 weeks post-infection in S19 vaccinated mice. Data are from one experiment.

We first measured CD44 expression as a marker of activation on CD4^+^ T cells after 4 weeks of vaccination in both CD4^Cre^*Bcl6*^fl/fl^ and *Bcl6*^fl/fl^ mice. We observed a significant decrease in the percentage of activated CD4^+^ T cells (CD44^+^CD4^+^) in vaccinated CD4^Cre^*Bcl6*^fl/fl^ mice when compared to vaccinated *Bcl6*^fl/fl^ counterparts (Fig. S3C). To further examine the role of *Bcl6* deficiency on CD4^+^ T-cell function, we performed RNA-seq on CD4^+^ T cells isolated from *Bcl6*^fl/fl^ and CD4^Cre^*Bcl6*^fl/fl^ mice after 4 weeks of vaccination and either 2 or 4 weeks of challenge. We identified a total of 1,565 differentially expressed genes (Log2FC > 0.5 and FDR < 0.05) in *Bcl6*-deficient CD4^+^ T cells, with 298 genes upregulated and 1,267 genes downregulated compared to their *Bcl6*^fl/fl^ CD4^+^ T-cell counterparts at 2 weeks post-challenge (Fig. 7A; Table S1). At 4 weeks post-challenge, 1,390 genes showed differential expression in *Bcl6*-deficient CD4^+^ T cells, with 994 genes upregulated and 396 genes downregulated (Fig. 7C; Table S2).

In our investigation, we identified a subset of genes (Fig. 7B, D and E) to highlight their possible impact within our vaccination/infection model. Of particular interest, the expression of *Ifng* remained unaltered between *Bcl6*-deficient and control CD4^+^ T cells at both time points. However, the expression of *Il10*, which is deleterious to host control of *Brucella* (40–43), and *Gata3*, which promotes IL-4 production (44), was significantly upregulated at both timepoints in CD4^+^ T cells lacking *Bcl6*. By contrast, *Bcl6* deficiency led to decreased expression of *Cxcr5*, which is associated with T_FH_, and *Tox2* (39)*,* which signals downstream of BCL6, at both timepoints. Collectively, these findings indicate *Bcl6* deficiency in CD4^+^ T cells is associated with a disrupted transcriptomic profile that could negatively impact S19 vaccine-mediated efficacy. The differential expression of some T-cell-related genes such as *Foxp3, Ccr7, Sell, Cd127*, *Cd44*, *Tnf, Tbx21, P2r × 7, Il22*, and *Il17re* varied between 2 and 4 weeks post-challenge. While CFU levels are similar in *Bcl6*^fl/fl^ and CD4^Cre^*Bcl6*^fl/fl^ mice after 4 weeks of vaccination and 2 weeks of challenge, after 4 weeks of challenge CFU levels are significantly higher in CD4^Cre^*Bcl6*^fl/fl^ mice (Fig. 6B and C). Therefore, differences in CFU levels between the two timepoints could impact the inflammatory environment and consequently alter gene expression by CD4^+^ T cells.

## DISCUSSION

Here, we reveal the multifaceted contributions of B cells in the context of S19 vaccination and *Brucella* infection. Our investigation demonstrates a phase-specific influence of B cells and antibodies on the effectiveness of S19 against brucellosis. Interestingly, the presence of B cells during the challenge phase is associated with a detrimental effect on vaccine efficacy (Fig. 1). By contrast, at the early stage of the challenge, we show IgM plays a pivotal role in protecting against *Brucella*; however, the role of antibodies diminishes as the infection progresses (Fig. 4). These observations indicate that the presence of B cells during vaccination is necessary for mounting a humoral response that impedes early dissemination of *B. melitensis*; however, the presence of B cell during the challenge phase may alter the immune response to create a favorable environment for the pathogen (16, 45, 46).

When investigating mechanisms by which B cells inhibit vaccine-mediated control of *Brucella*, we found S19-vaccinated mice lacking B-cell MHCII expression demonstrated enhanced resistance to *B. melitensis* during the later stage of challenge (Fig. 2B). This detrimental role aligns with a prior discovery from our laboratory, wherein a deleterious influence of B-cell MHCII expression during primary brucellosis was documented (18). The cumulative findings from our studies implicate B-cell antigen presentation as a limiting factor for both primary and vaccine-mediated immunity to *Brucella* (31).

Subsequently, our focus shifted toward the role of antibodies in S19-mediated efficacy. Notably, we observed S19-vaccinated *sIgM^−/−^/AID^−/−^* mice, which lack the capacity to produce both IgM and class-switched antibodies, demonstrated enhanced susceptibility to brucellosis 2 weeks after challenge (Fig. 4A and C). The concept of IgM offering a protective role against secondary brucellosis has been proposed by other researchers (19). In alignment with this perspective, our investigation using *sIgM^−/−^/AID^−/+^* animals confirmed IgM originating from S19 vaccination provides protection against brucellosis (Fig. 5A). IgM is a potent activator of complement (37), and unlike the reported deleterious role of complement in control of primary *B. abortus* infection (47), here we found complement depletion impaired vaccine-mediated immunity against *B. melitensis* (Fig. 5B). Therefore, in the future, we will investigate mechanisms by which complement activation alters control of primary and secondary *Brucella* infection.

As infection progressed, the role of antibody production in vaccine-mediated immunity was diminished. This change in antibody function across different infection stages could be attributed to the fact that following intraperitoneal inoculation, brucellae undergo a brief extracellular phase in the blood where they can be susceptible to specific circulating antibodies in immunized mice, thus reducing bacterial dissemination (48). Other intracellular pathogens that exhibit an *in vivo* extracellular stage are also susceptible to the impact of antibodies (49, 50). Nevertheless, the role of humoral immunity weakens as bacteria shift to an intracellular life where antibodies cannot access them, and host defenses predominantly rely on cellular immunity to control infection (51).

Our observation that IgM plays a greater role than IgG in S19-mediated efficacy against brucellosis was corroborated by studies in T_FH_-deficient mice which lack the ability to form germinal centers and undergo class switching (52) but can produce IgM (53). Our results indicate S19 vaccination continues to offer protection for T_FH_-deficient mice against brucellosis similar to *Bcl6*^fl/fl^ animals at the early stage of infection (Fig. 6A) confirming class switching is not essential for the efficacy of S19. Furthermore, it implies protective IgM elicited by S19 vaccination does not require germinal center formation. While class- switched antibodies do not appear essential for vaccine-mediated immunity, S19 vaccine efficacy does wane in CD4^Cre^*Bcl6*^fl/fl^ mice at later time points after challenge (Fig. 6B). In line with a prior report (38), our RNA-seq analysis (Fig. 6A through E) demonstrated an increased expression of *Il10* in *Bcl6*-deficient CD4 cells. This was of significance, as CD4^+^ T cell-derived IL-10 is deleterious to host control of *Brucella* (43). *Il10* is expressed by various immune cells including Th1, Th2, and Th17 cells, Treg cells, CD8^+^ T cells, and B cells (54). Although the specific *Il10*-producing cells in our model require further identification, the upregulation of *Gata3* and *Foxp3* in CD4^+^ T cells lacking *Bcl6* suggests that Th2 and Treg cells could be sources.

Collectively, this study sheds light on the protective role of IgM and the deleterious impact of B-cell MHCII expression in the context of live vaccine-mediated immunity against brucellosis. In addition, it underscores the detrimental role of B cells during the challenge phase and highlights the significance of *Bcl6* presence for CD4^+^ T-cell activation and the maintenance of a well-balanced inflammatory response throughout vaccination and challenge. Overall, our study improves our understanding of vaccine-mediated effector mechanisms against *Brucella* infection.

## MATERIALS AND METHODS

### Mice and animal use

Animal studies were conducted using sex-matched mice aged between 6 and 12 weeks of age. Mice were maintained in individually ventilated caging under high-efficiency particulate air-filtered barrier conditions with 12-h light and dark cycles within ABSL-3 facilities at the University of Missouri. Food and water were provided to animals *ad libitum*. All experiments were conducted in accordance with the University of Missouri Animal Care and Use Committee. Various immunodeficient and transgenic mouse strains on a C57BL/6J background (Table 1) were employed in this study. C57BL/6-Tg(IghelMD4)4Ccg/J (MD4), B6.129 × 1-*H2-Ab1^tm1Koni^*/J (*iAB^fl/fl^*B6.129S(FVB)-Bcl6tm1.1Dent/J (*Bcl6*^fl/fl^), and C57BL/6J (WT) mice were originally obtained from the Jackson Laboratory. *sIgM^−/−^*/*AID^−/−^* mice were a gift from Dr. Nicole Baumgarth at the University of California, Davis. *AID^−/−^* mice were originally generated at Kyoto University (55) and were bred to *sIgM^−/−^* mice at the Trudeau Institute (56). Mice with a singular deficiency in IgM secretion or class switching were generated by breeding *sIgM*^−/−^/*AID*^−/−^ mice with WT C57BL/6J mice (Table 1). B6.129P2(C)-Cd19tm1(cre)Cgn/J (CD19^Cre^), and B6.Cg-Tg(Cd4-cre)1Cwi/BfluJmice (CD4^Cre^) were a gift from Dr. Mark Daniels (University of Missouri). CD19^Cre^ animals were intercrossed with *iAB*^fl/fl^ animals to generate CD19^Cre^*iAB*^fl/fl^ mice. CD4^Cre^ mice were intercrossed with *Bcl6*^fl/fl^ mice to generate *Cd4*^Cre^*Bcl6*^fl/fl^ mice. Experiments involving the challenge of MD4, or CD19^Cre^*iAB*^fl/fl^ mice utilized HEL-negative or *iAB*^fl/fl^ litter mates as control animals, respectively. WT and *Bcl6*^fl/fl^ mice displayed similar phenotypes and were both used as controls for CD4^Cre^*Bcl6*^fl/fl^ animals.

**TABLE 1:** Mouse strains used in this study

Mouse strain	Phenotype
C57BL/6	Wild-type (WT) mice
MD4	Express BCR specific for hen egg lysozyme
*sIgM^−/−^/AID^−/−^*	Cannot secrete IgM or class switched antibodies
*sIgM^−/−^/AID^−/+^*	Cannot secrete IgM
*sIgM^−/+^/AID^−/−^*	Cannot secrete class-switched antibodies
*sIgM^−/+^/AID^−/+^*	Littermate control for *sIgM^−/+^/AID^−/−^* and *sIgM^−/−^/AID^−/+^* mice
CD19^Cre^*iAB*^fl/fl^	Lack B-cell MHCII expression
*iAB* ^fl/fl^	Control for CD19^Cre^*iAB*^fl/fl^ mice
CD4^cre^*Bcl6*^fl/fl^	Lack T follicular helper cells
*Bcl6* ^fl/fl^	Control for CD4^cre^*Bcl6*^fl/fl^ mice

### Bacteria and culture conditions

*Brucella melitensis* 16M was obtained from Montana State University (Bozeman, MT). All experiments with *B. melitensis* were performed in biosafety level 3 (BSL-3) facilities. The *Brucella abortus* S19 vaccine strain was obtained from the University of Wyoming (Laramie, WY). GFP-expressing S19 (GFP-S19) was generated by transforming pBBR1MCS6-Y (57) into S19 and followed by selection on agar with chloramphenicol (5 µg/mL). Bacteria were grown on Brucella agar (Becton Dickinson) at 37°C/5% CO_2_ before colonies were picked and cultured in Brucella broth overnight at 37°C in an orbital shaker. Challenge/vaccination doses were approximated by measurement of optical density at 600 nm and diluted using sterile Dulbecco’s phosphate-buffered saline (DPBS) (Thermofisher).

### Vaccination and challenge

Each animal received a single subcutaneous (s.c.) vaccination of 200 µL DPBS containing 2 × 10^5^ CFU *Brucella abortus* S19. The control group received DPBS only. Four or eight weeks post-vaccination, animals were challenged intraperitoneally (i.p.) with 1 × 10^5^ CFU *Brucella melitensis* 16M in a volume of 200 µL PBS. The vaccination and challenge doses were confirmed through 10-fold serial dilution and plating of inoculum onto Brucella agar plates.

### Immunoassay

Four weeks post-vaccination, peripheral blood samples were collected and centrifuged at 10,000 × *g* for 10 minutes at room temperature to obtain sera, which were stored at −80°C until further processing. An optimized indirect ELISA (31) was employed to measure antibody titers. To measure *Brucella*-specific antibody levels, ELISA plates were coated with 0.05 M carbonate/bicarbonate buffer containing heat-killed S19 at a concentration of 1 × 10^8^ CFU per well overnight at 4°C. For the standard curve, either unlabeled rat anti-mouse IgM (5 µg/mL) or goat anti-mouse IgG (0.5 µg/mL) (Southern Biotech) were used to coat IgM or IgG ELISA plates, respectively. The plates were then washed with 0.05% Tween and blocked with 1% BSA at 37°C. Diluted sera (1:50 or 1:2,000) or standards (mouse IgM or IgG) were added to the plates, which were maintained at room temperature for 2 h. After washing, HRP-conjugated secondary antibodies (1:1,000 for IgM or 1:4,000 for IgG) were added and incubated for an additional hour. TMB substrate (Invitrogen) was used to initiate the colorimetric reaction, and the reaction was stopped using 2N sulfuric acid. The plates were read at 450 nm using a SpectraMax Plus reader (Molecular Devices, San Jose, CA). All samples were tested in duplicate reactions. The limit of detection for anti-*Brucella* antibody was 30.9 pg/mL for IgM and 3.43 pg/mL for IgG.

### *In vivo* B-cell and complement depletion

We employed i.p. administration of 250 µg of anti-CD20 antibody (clone MB20-11, Southern Biotech) to deplete B cells from mice (58, 59). Animals were treated either on day 7 prior to vaccination or on day 21 post-vaccination (7 days before the challenge). Control animals received unlabeled mouse IgG as an isotype (Southern Biotech). For complement depletion, mice were treated i.p. with 10 µg of CVF (Complement Technology) twice at 4-h intervals both 1 day prior to challenge and 1 week post-challenge (60).

### Bacterial enumeration

At 2- or 4-week intervals after the challenge, the whole spleen was extracted from each mouse, weighed, and homogenized. Homogenates were 10-fold serially diluted and plated on erythritol-supplemented Brucella agar plates (1 mg/mL) to exclude S19 growth (61). Following 3–5 days of incubation at 37°C/5% CO_2_, colonies were counted.

### Flow cytometry

Spleens were homogenized and cell suspensions were filtered through a sterile 40-µm mesh following red blood cell lysis. Splenocytes were Fc blocked (2.4G2 Leinco) in fluorescence-activated cell-sorting (FACS) buffer (2% heat-inactivated fetal bovine serum [FBS] in DPBS) before extracellular staining. Blood samples were collected in 6.32 USP units per mL of sodium heparin (62), prior to RBC lysis and resuspension in FACS buffer and Fc block. After the Fc block, cells were stained with fluorochrome-conjugated mAbs: anti-CD4 (GK1.5 Biolegend), anti-CD44 (IM7 Biolegend), and anti-CD19 (1D3 Biolegend). Subsequently, the samples were washed and fixed with 4% paraformaldehyde (PFA) before being processed using a BD LSRFortessa X-20 flow cytometer. FlowJo software (Tree Star) was utilized to analyze the flow cytometry data.

### Confocal microscopy

Splenocytes were isolated from mice as described above and cultured in complete media (RPMI 1640 supplemented with HEPES, MEM, sodium pyruvate, and 10% FBS). A total of 3 × 10^6^ splenocytes per well were then added to an µ-Slide 8 Well chambered coverslip (ibidi) that had been pre-coated with 25 µg/mL Poly-L-lysine (Sigma). The cells were incubated for 1 h at 37°C/5% CO_2_ to ensure cell attachment, which was confirmed by inverted microscopy. After adherence, the cell culture media was removed and replaced with fresh media containing S19-GFP at a multiplicity of infection (MOI) of 500 and then incubated for 4 h at 37°C/5% CO_2_ before applying gentamicin (50 µg/mL) for an additional 30 minutes. Uninfected cells were used as a negative control.

For processing samples for confocal microscopy, the cells were washed with PBS, fixed with 2% PFA for 10 minutes, and blocked with 1% BSA for 30 minutes. The cells were then stained with APC-CD19 antibody (Biolegend) at a dilution of 1:100 in 1% BSA for 1 h at room temperature. Finally, the slide was washed three times to remove unbound antibodies and mounted using Calbiochem MOWIOL 4–88 anti-fade reagent (MilliporeSigma). The images were obtained using a TCS LeicaSP8 Confocal Microscope (Leica technologies) and analyzed using LAS X software (Leica technologies).

### Cell sorting

Splenocytes were isolated from mice as described above and CD4^+^ T cells were sorted using the EasySep Mouse CD4-Positive Selection Kit II (Stem Cell Technologies) following the manufacturer’s instructions. Aliquots of sorted cells were stained with anti-CD4 (GK1.5 Biolegend) to assess the cell purity by flow cytometry and the remaining samples were then preserved in RNAlater (Invitrogen) and kept at 4°C for subsequent RNA isolation. The purity of sorted splenic CD4^+^ T cells was confirmed to be ~90% by flow cytometry.

### RNA sequencing

RNA was extracted from sorted CD4^+^ T cells in RNAlater *via* the RNeasy Mini kit (Qiagen) following the manufacturer’s instructions. Poly A enriched stranded mRNA libraries were generated from extracted RNA and sequenced on a NovaSeq 6000 (Illumina) at the University of Missouri Genomics Technology Core. The RNA-seq data were analyzed using the OneStopRNAseq v1.0.0 server (https://mccb.umassmed.edu/OneStopRNAseq/)(63). Detailed parameter settings were followed as we recently described (64). Gencode.vM25.primary assembly was used as a mouse reference genome. Differentially expressed genes were filtered and selected using a false discovery rate (FDR) threshold of less than 0.05 and an absolute log2 fold change (log2FC) greater than 0.5. Heatmaps for selected genes were generated from the TPMs using the ClustVis server (https://biit.cs.ut.ee/clustvis/)(65).

### Statistical analyses

Statistical analyses were performed using GraphPad Prism software (version 9.2, GraphPad). Student *t*-tests were used to compare means between the two groups, with significance set at *P* ≤ 0.05. For comparisons involving three or more groups, one-way ANOVA was applied, followed by Turkey’s test for correction of multiple comparisons. Data are presented as average ±SD and statistical differences are indicated as *P ≤ 0.05; **P ≤ 0.01; ***P ≤ 0.001; ****P ≤ 0.0001; and ns, not significant.

## Data Availability

Data supporting the findings of this study are available in this paper, Supplementary information, or are available from the corresponding author upon request. The raw RNA-seq files used to generate the data in this manuscript have been deposited in the NCBI Gene Expression Omnibus (GSE243863).

## SUPPLEMENTAL MATERIAL

Figure S1 msphere.00750-23-s0001.tif Efficacy of B cell depletion.

Figure S2 msphere.00750-23-s0002.tif Antibody levels following S19 vaccination.

Figure S3 msphere.00750-23-s0003.tif Antibody levels and CD4+ T cell responses in TFH deficient mice.

Supplemental Material Legends msphere.00750-23-s0004.docx Legends for supplemental figures and tables.

Table S1 msphere.00750-23-s0005.xlsx Gene expression in CD4+ T cells from vaccinated mice at two weeks post-challenge.

Table S2 msphere.00750-23-s0006.xlsx Gene expression in CD4+ T cells in vaccinated mice at at four weeks post-challenge.

ASM does not own the copyrights to Supplemental Material that may be linked to, or accessed through, an article. The authors have granted ASM a non-exclusive, world-wide license to publish the Supplemental Material files. Please contact the corresponding author directly for reuse.

## References

[1] Franc KA, Krecek RC, Häsler BN, Arenas-Gamboa AM. 2018. Brucellosis remains a neglected disease in the developing world: a call for interdisciplinary action. BMC Public Health 18:125. doi:10.1186/s12889-017-5016-y 29325516 PMC5765637

[2] González-Espinoza G, Arce-Gorvel V, Mémet S, Gorvel JP. 2021. Brucella: reservoirs and niches in animals and humans. Pathogens 10:186. doi:10.3390/pathogens10020186 33572264 PMC7915599

[3] de Figueiredo P, Ficht TA, Rice-Ficht A, Rossetti CA, Adams LG. 2015. Pathogenesis and immunobiology of brucellosis. The American Journal of Pathology 185:1505–1517. doi:10.1016/j.ajpath.2015.03.003 25892682 PMC4450313

[4] Laine CG, Johnson VE, Scott HM, Arenas-Gamboa AM. 2023. Global estimate of human brucellosis incidence. Emerg Infect Dis 29:1789–1797. doi:10.3201/eid2909.230052 37610167 PMC10461652

[5] Pinn-Woodcock T, Frye E, Guarino C, Franklin-Guild R, Newman AP, Bennett J, Goodrich EL. 2023. A one-health review on brucellosis in the United States. J Am Vet Med Assoc 261:451–462. doi:10.2460/javma.23.01.0033 36862545

[6] Godfroid J. 2017. Brucellosis in livestock and wildlife: zoonotic diseases without pandemic potential in need of innovative one health approaches. Arch Public Health 75:34. doi:10.1186/s13690-017-0207-7 28904791 PMC5592711

[7] Nahas RS, Alsulami A, Lashkar MO, Thabit AK. 2022. Brucella epidydimo-orchitis successfully treated with dual oral drug regimen: a case report with differential diagnoses of malignancy and tuberculosis. Radiol Case Rep 17:3485–3489. doi:10.1016/j.radcr.2022.07.012 35912293 PMC9334919

[8] Yusufoğlu E, Kobat SG, Keser S. 2022. A rare cause of pediatric oculomotor nerve palsy: neurobrucellosis. Heliyon 8:e12134. doi:10.1016/j.heliyon.2022.e12134 36531647 PMC9747585

[9] Gokhale Y. 2022. *Brucella* arthritis, p 191–201. In Ravindran V, Santhanam S, Goyal M (ed), Rarer Arthropathies. Springer, Cham, Switzerland.

[10] Young EJ, Hasanjani Roushan MR, Shafae S, Genta RM, Taylor SL. 2014. Liver histology of acute brucellosis caused by Brucella melitensis. Hum Pathol 45:2023–2028. doi:10.1016/j.humpath.2014.07.007 25147098

[11] Reguera JM, Alarcón A, Miralles F, Pachón J, Juárez C, Colmenero JD. 2003. Brucella endocarditis: clinical, diagnostic, and therapeutic approach. Eur J Clin Microbiol Infect Dis 22:647–650. doi:10.1007/s10096-003-1026-z 14566576

[12] Khan MZ, Zahoor M. 2018. An overview of brucellosis in cattle and humans, and its serological and molecular diagnosis in control strategies. Trop Med Infect Dis 3:65. doi:10.3390/tropicalmed3020065 30274461 PMC6073575

[13] Pascual DW, Yang X, Wang H, Goodwin Z, Hoffman C, Clapp B. 2018. Alternative strategies for vaccination to brucellosis. Microbes Infect 20:599–605. doi:10.1016/j.micinf.2017.12.006 29287984 PMC6019614

[14] Darbandi A, Alamdary SZ, Koupaei M, Ghanavati R, Heidary M, Talebi M. 2022. Evaluation of immune responses to Brucella vaccines in mouse models: a systematic review. Front Vet Sci 9:903890. doi:10.3389/fvets.2022.903890 36118342 PMC9478790

[15] Pascual DW, Goodwin ZI, Bhagyaraj E, Hoffman C, Yang X. 2022. Activation of mucosal immunity as a novel therapeutic strategy for combating brucellosis. Front Microbiol 13:1018165. doi:10.3389/fmicb.2022.1018165 36620020 PMC9814167

[16] Goenka R, Parent MA, Elzer PH, Baldwin CL. 2011. B cell-deficient mice display markedly enhanced resistance to the intracellular bacterium Brucella abortus. J Infect Dis 203:1136–1146. doi:10.1093/infdis/jiq171 21451002

[17] Vitry M-A, De Trez C, Goriely S, Dumoutier L, Akira S, Ryffel B, Carlier Y, Letesson J-J, Muraille E. 2012. Crucial role of gamma interferon-producing CD4+ Th1 cells but dispensable function of CD8+ T cell, B cell, Th2, and Th17 responses in the control of Brucella melitensis infection in mice. Infect Immun 80:4271–4280. doi:10.1128/IAI.00761-12 23006848 PMC3497404

[18] Dadelahi AS, Lacey CA, Chambers CA, Ponzilacqua-Silva B, Skyberg JA. 2020. B cells inhibit CD4+ T cell-mediated immunity to Brucella infection in a major histocompatibility complex class II-dependent manner. Infect Immun 88:e00075-20. doi:10.1128/IAI.00075-20 32071068 PMC7171242

[19] Vitry M-A, Hanot Mambres D, De Trez C, Akira S, Ryffel B, Letesson J-J, Muraille E. 2014. Humoral immunity and CD4+ Th1 cells are both necessary for a fully protective immune response upon secondary infection with Brucella melitensis. J Immunol 192:3740–3752. doi:10.4049/jimmunol.1302561 24646742

[20] Demars A, Lison A, Machelart A, Van Vyve M, Potemberg G, Vanderwinden J-M, De Bolle X, Letesson J-J, Muraille E. 2019. Route of infection strongly impacts the host-pathogen relationship. Front Immunol 10:1589. doi:10.3389/fimmu.2019.01589 31354728 PMC6637429

[21] Jain L, Rawat M, Ramakrishnan S, Kumar B. 2017. Active immunization with Brucella abortus S19 phage lysate elicits serum IgG that protects guinea pigs against virulent B. abortus and protects mice by passive immunization. Biologicals 45:27–32. doi:10.1016/j.biologicals.2016.10.006 27913028

[22] Winter AJ, Duncan JR, Santisteban CG, Douglas JT, Adams LG. 1989. Capacity of passively administered antibody to prevent establishment of Brucella abortus infection in mice. Infect Immun 57:3438–3444. doi:10.1128/iai.57.11.3438-3444.1989 2509362 PMC259849

[23] Elzer PH, Jacobson RH, Jones SM, Nielsen KH, Douglas JT, Winter AJ. 1994. Antibody-mediated protection against Brucella abortus in BALB/C mice at successive periods after infection: variation between virulent strain 2308 and attenuated vaccine strain 19. Immunology 82:651–658. 7835931 PMC1414908

[24] Plommet M, Plommet AM. 1983. Immune serum-mediated effects on brucellosis evolution in mice. Infect Immun 41:97–105. doi:10.1128/iai.41.1.97-105.1983 6408007 PMC264748

[25] Verma S, Rawat M, Kumawat S, Qureshi S, Mohd G, Tiwari AK. 2018. Protective role of Brucella abortus specific murine antibodies in inhibiting systemic proliferation of virulent strain 544 in mice and guinea pig. Vet World 11:794–799. doi:10.14202/vetworld.2018.794-799 30034172 PMC6048087

[26] Adone R, Francia M, Pistoia C, Petrucci P, Pesciaroli M, Pasquali P. 2012. Protective role of antibodies induced by Brucella melitensis B115 against B. melitensis and Brucella abortus infections in mice. Vaccine 30:3992–3995. doi:10.1016/j.vaccine.2012.04.009 22521283

[27] Hoffmann EM, Houle JJ. 1995. Contradictory roles for antibody and complement in the interaction of Brucella abortus with its host. Crit Rev Microbiol 21:153–163. doi:10.3109/10408419509113538 8845060

[28] van Straten M, Bardenstein S, Keningswald G, Banai M. 2016. Brucella abortus S19 vaccine protects dairy cattle against natural infection with Brucella melitensis. Vaccine 34:5837–5839. doi:10.1016/j.vaccine.2016.10.011 27771184

[29] Hensel ME, Chaki SP, Stranahan L, Gregory AE, van Schaik EJ, Garcia-Gonzalez DG, Khalaf O, Samuel JE, Arenas-Gamboa AM. 2020. Intratracheal inoculation with Brucella melitensis in the pregnant guinea pig is an improved model for reproductive pathogenesis and vaccine studies. Infect Immun 88:e00204-20. doi:10.1128/IAI.00204-20 32690632 PMC7504952

[30] Bosseray N, Plommet M. 1990. Brucella suis S2, Brucella melitensis Rev. 1 and Brucella abortus S19 living vaccines: residual virulence and immunity induced against three Brucella species challenge strains in mice. Vaccine 8:462–468. doi:10.1016/0264-410x(90)90247-j 2123586

[31] Dadelahi AS, Abushahba MFN, Ponzilacqua-Silva B, Chambers CA, Moley CR, Lacey CA, Dent AL, Skyberg JA, Tsolis RM. 2023. Interactions between B cells and T follicular regulatory cells enhance susceptibility to Brucella infection independent of the anti-Brucella humoral response. PLoS Pathog 19:e1011672. doi:10.1371/journal.ppat.1011672 37721965 PMC10538787

[32] Hanot Mambres D, Machelart A, Potemberg G, De Trez C, Ryffel B, Letesson J-J, Muraille E. 2016. Identification of immune effectors essential to the control of primary and secondary intranasal infection with Brucella melitensis in mice. J Immunol 196:3780–3793. doi:10.4049/jimmunol.1502265 27036913

[33] Grilló M-J, Blasco JM, Gorvel JP, Moriyón I, Moreno E. 2012. What have we learned from brucellosis in the mouse model? Vet Res 43:29. doi:10.1186/1297-9716-43-29 22500859 PMC3410789

[34] Barroso M, Tucker H, Drake L, Nichol K, Drake JR. 2015. Antigen-B cell receptor complexes associate with intracellular major histocompatibility complex (MHC) class II molecules. J Biol Chem 290:27101–27112. doi:10.1074/jbc.M115.649582 26400081 PMC4646406

[35] Goodnow CC, Crosbie J, Adelstein S, Lavoie TB, Smith-Gill SJ, Brink RA, Pritchard-Briscoe H, Wotherspoon JS, Loblay RH, Raphael K, Trent RJ, Basten A. 1988. Altered immunoglobulin expression and functional silencing of self-reactive B lymphocytes in transgenic mice. Nature 334:676–682. doi:10.1038/334676a0 3261841

[36] Kumazaki K, Tirosh B, Maehr R, Boes M, Honjo T, Ploegh HL. 2007. AID−/−μs−/− mice are agammaglobulinemic and fail to maintain B220−CD138+ plasma cells. J Immunol 178:2192–2203. doi:10.4049/jimmunol.178.4.2192 17277124

[37] Gong S, Ruprecht RM. 2020. Immunoglobulin M: an ancient antiviral weapon – rediscovered. Front Immunol 11:1943. doi:10.3389/fimmu.2020.01943 32849652 PMC7432194

[38] Hollister K, Kusam S, Wu H, Clegg N, Mondal A, Sawant DV, Dent AL. 2013. Insights into the role of Bcl6 in Follicular Th cells using a new conditional mutant mouse model. J Immunol 191:3705–3711. doi:10.4049/jimmunol.1300378 23980208 PMC3783642

[39] Choi J, Crotty S. 2021. Bcl6-mediated transcriptional regulation of follicular helper T cells (TFH). Trends Immunol 42:336–349. doi:10.1016/j.it.2021.02.002 33663954 PMC8021443

[40] Fernandes DM, Jiang X, Jung JH, Baldwin CL. 1996. Comparison of T cell cytokines in resistant and susceptible mice infected with virulent Brucella abortus strain 2308. FEMS Immunol Med Microbiol 16:193–203. doi:10.1111/j.1574-695X.1996.tb00136.x 9116636

[41] Fernandes DM, Baldwin CL. 1995. Interleukin-10 downregulates protective immunity to Brucella abortus. Infect Immun 63:1130–1133. doi:10.1128/iai.63.3.1130-1133.1995 7868238 PMC173122

[42] Corsetti PP, de Almeida LA, Carvalho NB, Azevedo V, Silva TMA, Teixeira HC, Faria AC, Oliveira SC, Alves-Filho JC. 2013. Lack of endogenous IL-10 enhances production of proinflammatory cytokines and leads to Brucella abortus clearance in mice. PLoS ONE 8:e74729. doi:10.1371/journal.pone.0074729 24069337 PMC3775771

[43] Xavier MN, Winter MG, Spees AM, Nguyen K, Atluri VL, Silva TMA, Bäumler AJ, Müller W, Santos RL, Tsolis RM. 2013. CD4+ T cell-derived IL-10 promotes Brucella abortus persistence via modulation of macrophage function. PLoS Pathog 9:e1003454. doi:10.1371/journal.ppat.1003454 23818855 PMC3688575

[44] Zhu J, Yamane H, Cote-Sierra J, Guo L, Paul WE. 2006. GATA-3 promotes Th2 responses through three different mechanisms: induction of Th2 cytokine production, selective growth of Th2 cells and inhibition of Th1 cell-specific factors. Cell Res 16:3–10. doi:10.1038/sj.cr.7310002 16467870

[45] Goenka R, Guirnalda PD, Black SJ, Baldwin CL. 2012. B lymphocytes provide an infection niche for intracellular bacterium Brucella abortus. J Infect Dis 206:91–98. doi:10.1093/infdis/jis310 22561364 PMC3415929

[46] García-Gil A, Lopez-Bailon LU, Ortiz-Navarrete V. 2019. Beyond the antibody: B cells as a target for bacterial infection. J Leukoc Biol 105:905–913. doi:10.1002/JLB.MR0618-225R 30657607

[47] González-Espinoza G, Barquero-Calvo E, Lizano-González E, Alfaro-Alarcón A, Arias-Gómez B, Chaves-Olarte E, Lomonte B, Moreno E, Chacón-Díaz C, Roy CR. 2018. Depletion of complement enhances the clearance of Brucella abortus in mice. Infect Immun 86:e00567-18. doi:10.1128/IAI.00567-18 30082480 PMC6204725

[48] Vitry M-A, Hanot Mambres D, Deghelt M, Hack K, Machelart A, Lhomme F, Vanderwinden J-M, Vermeersch M, De Trez C, Pérez-Morga D, Letesson J-J, Muraille E, Morrison RP. 2014. Brucella melitensis invades murine erythrocytes during infection. Infect Immun 82:3927–3938. doi:10.1128/IAI.01779-14 25001604 PMC4187840

[49] Silva MT, Silva Pestana NT. 2013. The in vivo extracellular life of facultative intracellular bacterial parasites: role in pathogenesis. Immunobiology 218:325–337. doi:10.1016/j.imbio.2012.05.011 22795971

[50] Casadevall A. 2003. Antibody-mediated immunity against intracellular pathogens: two-dimensional thinking comes full circle. Infect Immun 71:4225–4228. doi:10.1128/IAI.71.8.4225-4228.2003 12874297 PMC166024

[51] Skendros P, Boura P. 2013. Immunity to brucellosis. Rev Sci Tech OIE 32:137–147. doi:10.20506/rst.32.1.2190 23837372

[52] Nurieva RI, Chung Y, Martinez GJ, Yang XO, Tanaka S, Matskevitch TD, Wang YH, Dong C. 2009. Bcl6 mediates the development of T follicular helper cells. Science 325:1001–1005. doi:10.1126/science.1176676 19628815 PMC2857334

[53] Lee SK, Rigby RJ, Zotos D, Tsai LM, Kawamoto S, Marshall JL, Ramiscal RR, Chan TD, Gatto D, Brink R, Yu D, Fagarasan S, Tarlinton DM, Cunningham AF, Vinuesa CG. 2011. B cell priming for extrafollicular antibody responses requires Bcl-6 expression by T cells. J Exp Med 208:1377–1388. doi:10.1084/jem.20102065 21708925 PMC3135363

[54] Murakami M, Hirano T. 2012. The molecular mechanisms of chronic inflammation development. Front Immunol 3:323. doi:10.3389/fimmu.2012.00323 23162547 PMC3498841

[55] Muramatsu M, Kinoshita K, Fagarasan S, Yamada S, Shinkai Y, Honjo T. 2000. Class switch recombination and hypermutation require activation-induced cytidine deaminase (AID), a potential RNA editing enzyme. Cell 102:553–563. doi:10.1016/s0092-8674(00)00078-7 11007474

[56] Carragher DM, Kaminski DA, Moquin A, Hartson L, Randall TD. 2008. A novel role for non-neutralizing antibodies against nucleoprotein in facilitating resistance to influenza virus. J Immunol 181:4168–4176. doi:10.4049/jimmunol.181.6.4168 18768874 PMC2590646

[57] Murphy E, Robertson GT, Parent M, Hagius SD, Roop RM, Elzer PH, Baldwin CL. 2002. Major histocompatibility complex class I and II expression on macrophages containing a virulent strain of Brucella abortus measured using green fluorescent protein-expressing Brucellae and flow cytometry. FEMS Immunol Med Microbiol 33:191–200. doi:10.1111/j.1574-695X.2002.tb00590.x 12110481

[58] Liu CL, Ye P, Lin J, Butts CL, Miao CH. 2014. Anti-CD20 as the B-cell targeting agent in a combined therapy to modulate anti-factor VIII immune responses in hemophilia A inhibitor mice. Front Immunol 4:502. doi:10.3389/fimmu.2013.00502 24432019 PMC3881000

[59] Hamaguchi Y, Uchida J, Cain DW, Venturi GM, Poe JC, Haas KM, Tedder TF. 2005. The peritoneal cavity provides a protective niche for B1 and conventional B lymphocytes during anti-CD20 immunotherapy in mice. J Immunol 174:4389–4399. doi:10.4049/jimmunol.174.7.4389 15778404

[60] Zhang G, Peng Y, Schoenlaub L, Elliott A, Mitchell W, Zhang Y. 2013. Formalin-Inactivated Coxiella burnetii phase I vaccine-induced protection depends on B cells to produce protective IgM and IgG. Infect Immun 81:2112–2122. doi:10.1128/IAI.00297-13 23545296 PMC3676018

[61] Grilló MJ, Manterola L, de Miguel MJ, Muñoz PM, Blasco JM, Moriyón I, López-Goñi I. 2006. Increases of efficacy as vaccine against Brucella abortus infection in mice by simultaneous inoculation with avirulent smooth bvrS/bvrR and rough wbkA mutants. Vaccine 24:2910–2916. doi:10.1016/j.vaccine.2005.12.038 16439039

[62] Abushahba MF, Dadelahi AS, Lemoine EL, Skyberg JA, Vyas S, Dhoble S, Ghodake V, Patravale VB, Adamovicz JJ. 2023. Safe subunit green vaccines confer robust immunity and protection against mucosal Brucella infection in mice. Vaccines (Basel) 11:546. doi:10.3390/vaccines11030546 36992130 PMC10051820

[63] Li R, Hu K, Liu H, Green MR, Zhu LJ. 2020. OnestopRNAseq: a web application for comprehensive and efficient analyses of RNA-Seq data. Genes (Basel) 11:1165. doi:10.3390/genes11101165 33023248 PMC7650687

[64] Moley CR, Chambers CA, Dadelahi AS, Ponzilacqua-Silva B, Abushahba MFN, Lacey CA, Franklin CL, Skyberg JA. 2023. Innate lymphoid cells and interferons limit neurologic and articular complications of brucellosis. Am J Pathol 193:1170–1184. doi:10.1016/j.ajpath.2023.05.006 37263343 PMC10477959

[65] Metsalu T, Vilo J. 2015. Clustvis: a web tool for visualizing clustering of multivariate data using principal component analysis and heatmap. Nucleic Acids Res 43:W566–W570. doi:10.1093/nar/gkv468 25969447 PMC4489295

