# Supplementary figures and images for: Contrasting roles for IgM and B-cell MHCII expression in Brucella abortus S19 vaccine-mediated efficacy against B. melitensis infection
Source: mSphere. 2024 Feb 13;9(3):e00750-23. doi: 10.1128/msphere.00750-23 (PMC10964430; doi:10.1128/msphere.00750-23)

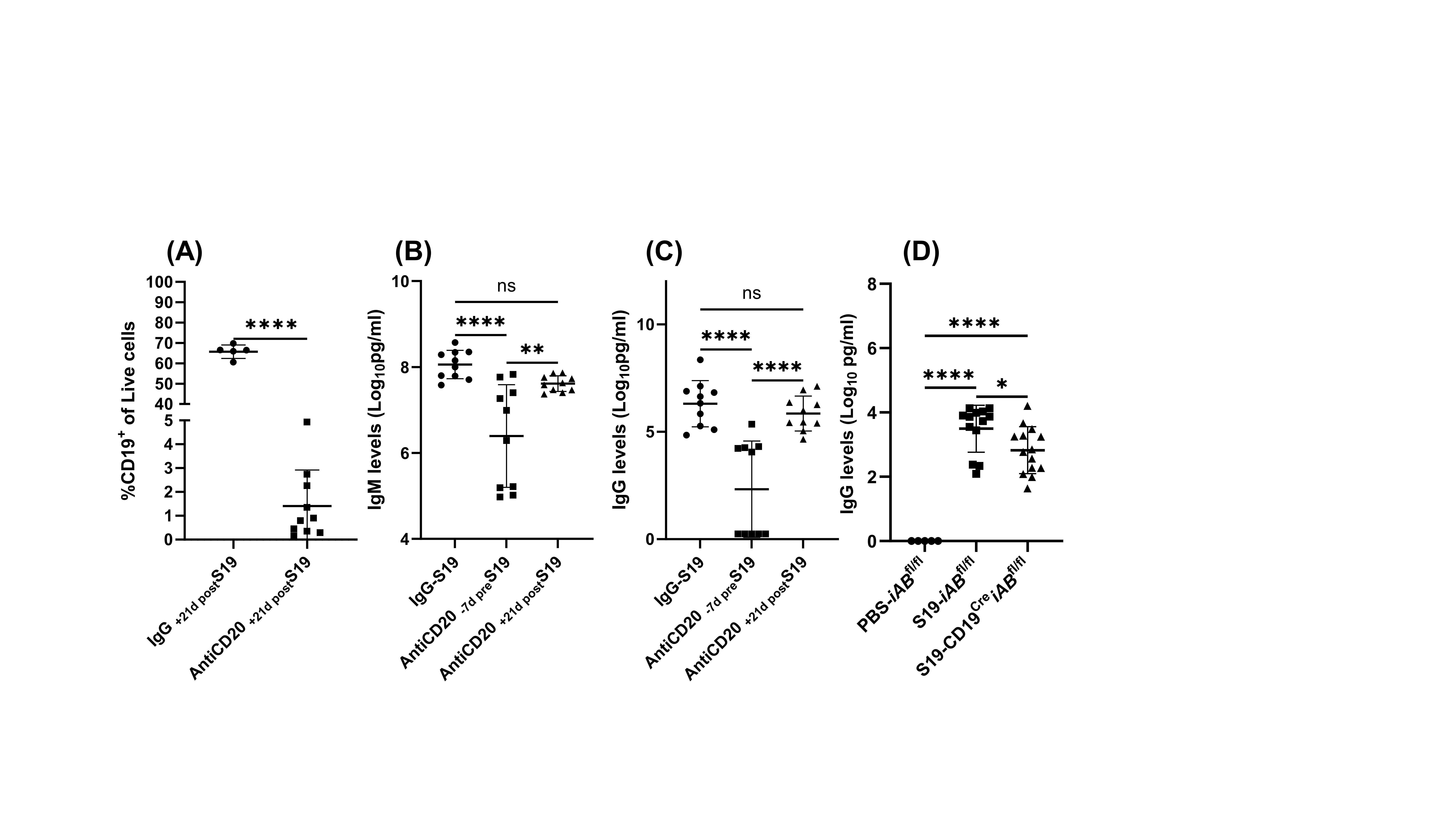

Supplement: Figure S1 — Efficacy of B cell depletion. [file msphere.00750-23-s0001.tif]

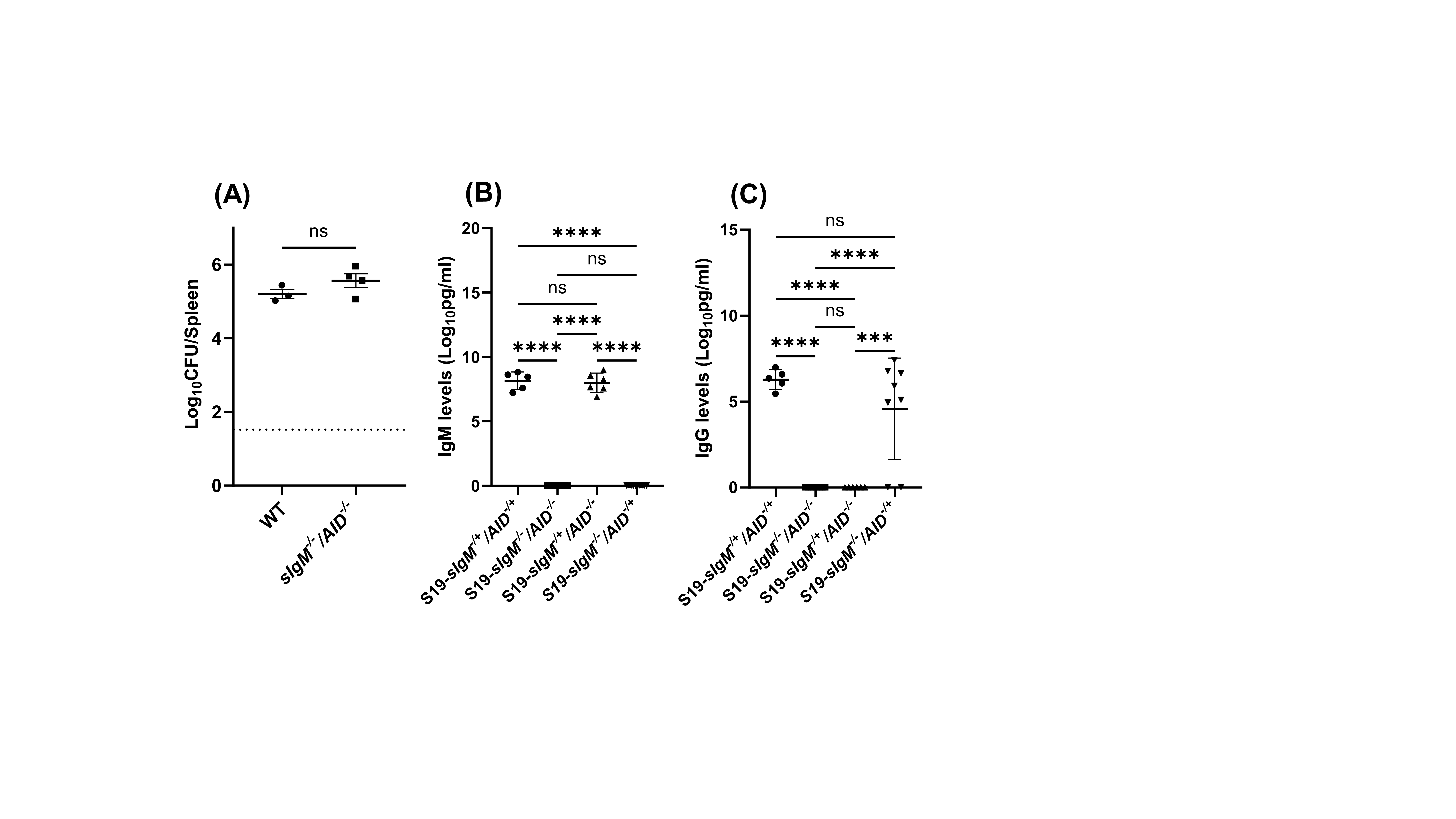

Supplement: Figure S2 — Antibody levels following S19 vaccination. [file msphere.00750-23-s0002.tif]

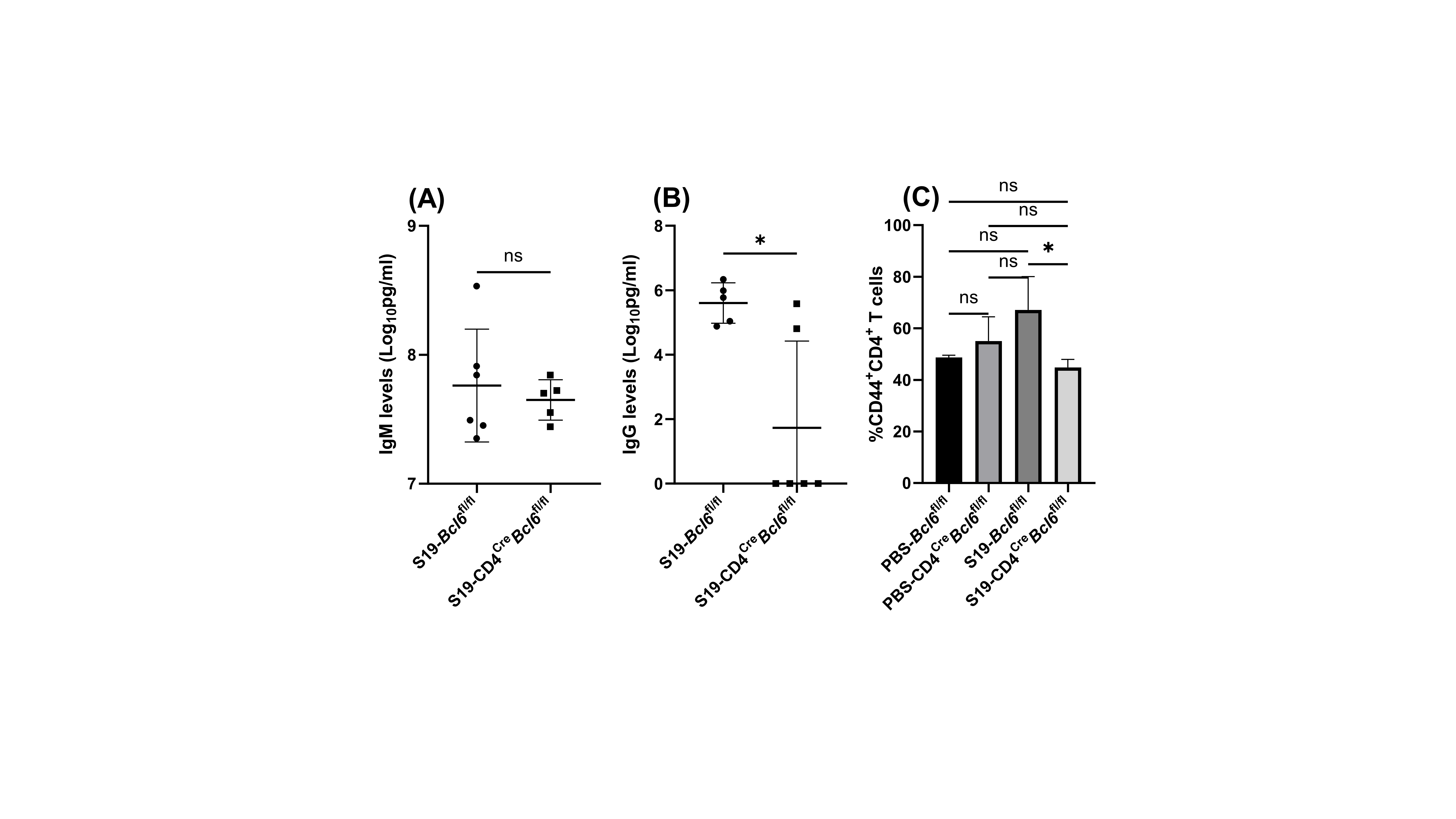

Supplement: Figure S3 — Antibody levels and CD4+ T cell responses in TFH deficient mice. [file msphere.00750-23-s0003.tif]
