# Supplementary material for: Contrasting roles for IgM and B-cell MHCII expression in Brucella abortus S19 vaccine-mediated efficacy against B. melitensis infection
Source: mSphere. 2024 Feb 13;9(3):e00750-23. doi: 10.1128/msphere.00750-23 (PMC10964430; doi:10.1128/msphere.00750-23)
Supplement: Supplemental Material Legends — Legends for supplemental figures and tables. [file msphere.00750-23-s0004.docx]

**Supplementary Material Legends:**

**Figure S1. Efficacy of B cell depletion.**  **(A)** WT mice were vaccinated s.c. with 2 x 10^5^ CFUs of S19 and at 21 days post vaccination (one week before challenge), mice were treated i.p. with anti-CD20 antibody or IgG. 7 days later, blood samples were processed for flow cytometry. (**B** and **C**) WT mice were vaccinated s.c. with 2 x 10^5^ CFUs of S19. anti-CD20 antibody was injected i.p to mice either 7 days before vaccination (referred in the figures as Anti-CD20 _-7pre_S19) or 21 days after vaccination (referred in the figures as Anti-CD20 _+21post_S19). 28 days after vaccination, anti-*Brucella* IgM (**B**) and IgG (**C**) levels were measured in sera. **(D)** CD19^Cre^*iAB*^fl/fl^ and *iAB*^fl/fl^ mice were treated with PBS or vaccinated s.c. with 2 x 10^5^ CFUs of S19. 28 days after vaccination, anti-*Brucella* IgG levels were measured in sera (**D**).

**Figure S2.** **Antibody levels following S19 vaccination. (A)** Splenic *B. melitensis* burdens in naïve *sIgM^-/-^/AID^-/-^* and WT mice (n=3-4 mice/per group) measured two weeks after challenge. Anti-*Brucella* IgM and IgG levels (**B-C)** were measured in sera obtained from *sIgM^-/+^/AID^-/+^* *sIgM^-/-^/AID^-/-^*, *sIgM^-/+^/AID^-/-^* and *sIgM^-/-^/AID^-/+^* mice on day 28 after s.c. vaccination with 2 x 10^5^ CFUs of S19 (prior to challenge).

**Figure S3. Antibody levels and CD4^+^ T cell responses in T_FH_ deficient mice.** (A & B) Sera were obtained from animals on day 28 s.c. postvaccination with 2 x 10^5^ CFUs of S19 (prior to challenge) and analyzed by ELISA for *Brucella*- specific IgM and IgG antibody levels. **(C)** Mice were s.c vaccinated with 2 x 10^5^ CFUs of S19 vaccine. On day 28 post S19 vaccination, spleens were processed from CD4^Cre^*Bcl6*^fl/fl^ and *Bcl6*^fl/fl^ mice and CD44 expression was assessed on CD4^+^ T cells by flow cytometry.

**Supplementary Table 1. Differential gene expression in CD4^+^ T cells from S19 vaccinated *Bcl6*^fl/fl^ and CD4^Cre^*Bcl6*^fl/fl^ mice two weeks after challenge with B. melitensis.** Mice were s.c vaccinated with 2 x 10^5^ CFUs of S19 vaccine and were challenged 4-weeks later with 1 x 10^5^ CFUs i.p. of *B. melitensis* 16M. Two weeks post-challenge, CD4^+^ T cells were purified from spleens and RNA was extracted for RNA-Seq analysis.

**Supplementary Table 2. Differential gene expression in CD4^+^ T cells from S19 vaccinated *Bcl6*^fl/fl^ and CD4^Cre^*Bcl6*^fl/fl^ mice four weeks after challenge with B. melitensis.** Mice were s.c vaccinated with 2 x 10^5^ CFUs of S19 vaccine and were challenged 4-weeks later with 1 x 10^5^ CFUs i.p. of *B. melitensis* 16M. Four weeks post-challenge, CD4^+^ T cells were purified from spleens and RNA was extracted for RNA-Seq analysis.
